# Massive Losses of Taste Receptor Genes in Toothed and Baleen Whales

**DOI:** 10.1093/gbe/evu095

**Published:** 2014-05-06

**Authors:** Ping Feng, Jinsong Zheng, Stephen J. Rossiter, Ding Wang, Huabin Zhao

**Affiliations:** ^1^Department of Zoology, College of Life Sciences, Wuhan University, China; ^2^Institute of Hydrobiology, Chinese Academy of Sciences, Wuhan, China; ^3^School of Biological and Chemical Sciences, Queen Mary, University of London, United Kingdom

**Keywords:** taste receptor, evolution, whales, pseudogenization, diet

## Abstract

Taste receptor genes are functionally important in animals, with a surprising exception in the bottlenose dolphin, which shows extensive losses of sweet, umami, and bitter taste receptor genes. To examine the generality of taste gene loss, we examined seven toothed whales and five baleen whales and sequenced the complete repertoire of three sweet/umami (*T1R*s) and ten bitter (*T2R*s) taste receptor genes. We found all amplified *T1R*s and *T2R*s to be pseudogenes in all 12 whales, with a shared premature stop codon in 10 of the 13 genes, which demonstrated massive losses of taste receptor genes in the common ancestor of whales. Furthermore, we analyzed three genome sequences from two toothed whales and one baleen whale and found that the sour taste marker gene *Pkd2l1* is a pseudogene, whereas the candidate salty taste receptor genes are intact and putatively functional. Additionally, we examined three genes that are responsible for taste signal transduction and found the relaxation of functional constraints on taste signaling pathways along the ancestral branch leading to whales. Together, our results strongly suggest extensive losses of sweet, umami, bitter, and sour tastes in whales, and the relaxation of taste function most likely arose in the common ancestor of whales between 36 and 53 Ma. Therefore, whales represent the first animal group to lack four of five primary tastes, probably driven by the marine environment with high concentration of sodium, the feeding behavior of swallowing prey whole, and the dietary switch from plants to meat in the whale ancestor.

## Introduction

Taste perception is fundamental for the survival of animals ranging from insects to mammals (Yarmolinsky et al. 2009). The sense of taste is highly specialized to sense nutritious or harmful compounds in potential food sources and is therefore essential to trigger or regulate feeding behaviors in animals (Bachmanov and Beauchamp 2007). Animals are commonly believed to have five primary taste modalities: Sweet, umami, bitter, sour, and salty in mammals; and sweet, bitter, water, carbonation, and salty in insects (Bachmanov and Beauchamp 2007; Yarmolinsky et al. 2009). Although mammals and insects diverged from their common ancestor approximately 900 Ma (Hedges et al. 2004), both groups of animals share the same fundamental principles for encoding taste information (Yarmolinsky et al. 2009).

Taste is activated by the physical interactions between tastant molecules from the external environment and taste receptors, the latter of which have been decoded in the last decade (Bachmanov and Beauchamp 2007; Yarmolinsky et al. 2009). Vertebrate taste receptors are either ion channels (sour and salty) or two families of G protein-coupled receptors (T1Rs for sweet/umami and T2Rs for bitter), whereas gustatory receptors (GRs) are primarily responsible for taste in insects. Because of the essential roles of taste, taste receptor genes are believed to be indispensable from insects to mammals. For example, the sweet/umami taste receptor gene repertoire (*T1R*s) generally contains three members across mammals; the number of *GR*s remains similar in most insects (Nei et al. 2008). Although vertebrate bitter taste receptor genes (*T2R*s) vary remarkably in number from 3 in chicken to 49 in frog (Shi and Zhang 2006), the bitter taste is still required to detect toxins in food sources for these animals. However, numerous pseudogenizations of taste receptor genes have been discovered in animals along with an increasing number of available genome sequences. For example, the sweet taste receptor gene (*T1R2*) is lost in the chicken, zebra finch, cat, vampire bats, and western clawed frog (Li et al. 2005; Shi and Zhang 2006; Zhao et al. 2010); the umami taste receptor gene *T1R1* is lost in the giant panda, western clawed frog, and bats (Shi and Zhang 2006; Zhao, Yang, et al. 2010; Zhao et al. 2012). Strikingly, the draft dolphin genome (2.59× coverage) lacks intact genes responsible for sweet, umami, and bitter tastes (Jiang et al. 2012). The lack of bitter taste is particularly unexpected because natural toxins typically taste bitter, and bitter taste thus represents an important natural defense against the ingestion of poisonous chemicals from the external environment (Glendinning 1994).

In addition to taste receptors, signaling pathways downstream of taste receptors are also essential for taste function. Specifically, three ion channels (TRPM5, PLCβ2, and CALHM1) have been identified as key components of taste signal transduction to detect sweet, umami, and bitter tastes (Zhang et al. 2003; Taruno et al. 2013). TRPM5 (Transient receptor potential cation channel subfamily M member 5), a taste-specific TRP ion channel, is coexpressed with T1R and T2R cells in taste buds, and mice deficient for *Trpm5* exhibit abolished or severely reduced sensitivity to sweet, umami, and bitter stimuli (Zhang et al. 2003; Damak et al. 2006). Similar to TRPM5, PLCβ2 (phospholipase Cβ2) shares overlapping expression patterns with TRPM5, and its knockout mice show a selective and complete loss of sweet, umami, and bitter responses (Zhang et al. 2003). CALHM1 (calcium homeostasis modulator 1) contributes to the neurotransmission of taste stimuli; the loss of CALHM1 rendered severely impaired responses to sweet, umami, and bitter tastants (Taruno et al. 2013). Thus, *Trpm5*, *Plcβ2,* and *Calhm1* likely undergo the relaxation of selective constraints if sweet, umami, and bitter tastes have been lost for a considerable amount of time.

**Fig. 1.—:**
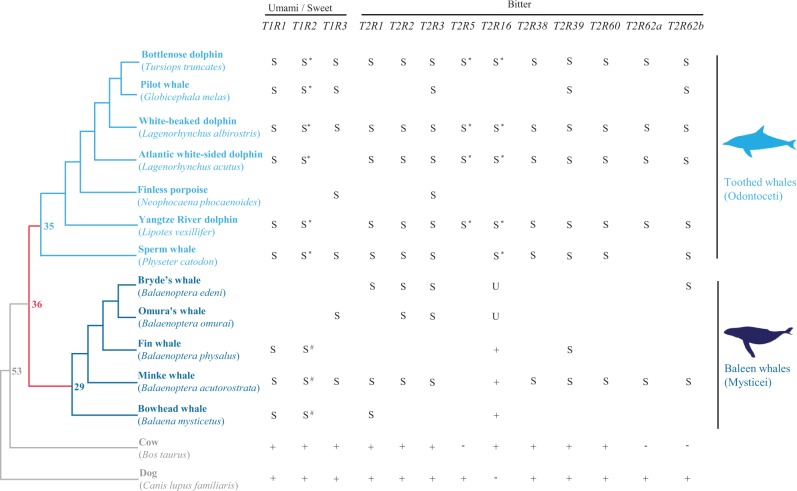
The species tree of whales with common mutations of *T1R*s and *T2R*s. The tree topology and divergence dates follow previous studies (Hasegawa et al. 2003; McGowen et al. 2009). Branch lengths are not drawn to scale; the numbers at the nodes are the divergence times in millions of years. *T1R1* and *T1R3* encode the umami taste receptor, *T1R2* and *T1R3* encode sweet taste receptors, and *T2R*s confer bitter taste. For each gene, shared frame-shifting indels or premature stop codons across toothed and baleen whales are indicated by “S,” shared frame-shifting mutations in one of the two major lineages of whales are indicated with “S*” (toothed whales) or “S^#^” (baleen whales), and unshared ones are shown with “U.” Additionally, “+” indicates the presence of an intact ORF, “−” denotes the absence from the genome, while no signs in any gene indicate no amplifications despite numerous attempts.

To examine the generality of taste loss in whales and determine when whales lost tastes, we sequenced *T1R*s and *T2R*s in 11 species of whales: The bottlenose dolphin (*Tursiops truncates*), pilot whale (*Globicephala melas*), white-beaked dolphin (*Lagenorhynchus albirostris*), Atlantic white-sided dolphin (*L. acutus*), finless porpoise (*Neophocaena phocaenoides*), sperm whale (*Physeter catodon*), Bryde's whale (*Balaenoptera edeni*), Omura's whale (*B. omurai*), fin whale (*B. physalus*), minke whale (*B. acutorostrata*), and bowhead whale (*Balaena mysticetus*) (fig. 1). Our sample contained representatives from both major lineages of whales: Odontoceti (toothed whales) and Mysticeti (baleen whales) (McGowen et al. 2009) (fig. 1). We included three sweet/umami taste receptor genes (*T1R1-3*) and all ten bitter taste receptor genes (*T2R1-3, T2R5, T2R16, T2R38-39, T2R60,* and *T2R62a-62 b*) identified from the draft dolphin genome (Jiang et al. 2012). We also examined the draft genome sequences of the bottlenose dolphin, Yangtze River dolphin (Zhou et al. 2013), and minke whale (Yim et al. 2014) to identify genes potentially responsible for sour and salty tastes. To test whether taste signaling pathways have been relaxed from selective constraints because of taste loss, we analyzed the *Calhm1*, *Plcβ2,* and *Trpm5* sequences from several whales in comparison with other mammals. We show that the sweet, umami, bitter, and sour taste receptor genes are pseudogenized, whereas salty taste receptor genes are evolutionarily conserved in toothed and baleen whales. We further show the relaxation of selective constraints on taste signaling pathways along the ancestral branch leading to whales (*Plcβ2* and *Calhm1*) or after the divergence of whales (*Trpm5*).

## Materials and Methods

### Polymerase Chain Reaction and DNA Sequencing

By searching the draft genome sequence of the bottlenose dolphin (2.59X), we identified all three members of *T1R*s, all ten members of *T2R*s, and *Calhm1*. These sequences were used to design a suite of primers (supplementary table S1, Supplementary Material online), which were employed to amplify the 14 genes in other whales. Genetic material was obtained from the Institute of Hydrobiology, Chinese Academy of Sciences, and the marine mammal tissue archive held at Zoological Society of London. Our samples contained six species of toothed whales (bottlenose dolphin, pilot whale, white-beaked dolphin, Atlantic white-sided dolphin, finless porpoise, and sperm whale) and five species of baleen whales (Bryde's whale, Omura's whale, fin whale, minke whale, and bowhead whale) (fig. 1). The genomic DNAs were isolated using the Qiagen DNeasy kit. The polymerase chain reaction (PCR) mixtures (30 μl) contained 0.5 μl genomic DNA (10 ng/μl), 15 μl of 2× PCR solution (Takara Premix Taq), and 1.2 μl of each primer (10 μM). When the above PCRs did not work, we used the following PCR mixtures (25 μl): 0.5 μl genomic DNA (10 ng/μl), 2.5 μl of 10× PCR buffer, 2.5 μl dNTPs, 1.5 μl MgSO_4_, 0.75 μl of each primer (10 μM), 0.5 μl (1U/ μl) of KOD-Plus-Neo DNA polymerase (Toyobo), and 16 μl H_2_O. All PCRs were conducted on a BioRad T100 Thermal Cycler under the touchdown conditions as follows: 5 min of initial denaturation, 15 cycles of denaturation at 95 °C for 30 s, 65 °C for 30 s (the temperature was decreased by 1 °C per cycle), extension at 72 °C for 60 s; followed by 25 cycles with 95 °C for 30 s, 50 °C for 30 s, 72 °C for 60 s; and a final extension at 72 °C for 5 min. Sequencing reactions were performed directly from both strands with the same primer sets as those used for the PCR amplifications. When the direct sequencing did not work, the PCR products were cloned into the pMD19-T vector (Takara) and transformed to DH-5α competent cells. Three to five positive clones of each PCR product were screened and sequenced. All newly generated sequences by PCRs were submitted to GenBank under accession numbers KJ547495–KJ547591.

### Data Mining from Mammalian Genome Sequences

For the identification of multiple-exon genes, we used mouse genes as query sequences to conduct TBLASTN searches (Altschul et al. 1990) to identify sour taste marker genes (*Pkd2l1*and *Pkd1l3*), the candidate salty taste receptor genes (*Scnn1a*, *Scnn1b,* and *Scnn1g*), and three taste signaling pathway genes (*Calhm1*, *Trpm5,* and *Plcβ2*) from three publicly available genome sequences of whales (bottlenose dolphin, Yangtze River dolphin, and mink whale). To validate the BLAST results, we retrieved the matched scaffolds from genome sequences, and the exon/intron structures were determined using a combination of GeneWise (Birney et al. 2004) and NCBI-BLAST2 (Altschul et al. 1997) programs. For the identification of single-exon genes (*T2R*s), we used all *T2R*s from human, rat, dog, and chicken as queries to TBLASTN the three whale genomes following a previous study (Shi and Zhang 2006) and confirmed the presence of seven transmembrane domains using the TMHMM method (Sonnhammer et al. 1998). All candidate genes were verified by the best hits with known genes of interest using BLASTP searches against the entire GenBank (Shi and Zhang 2006). All sequences culled from the three whale genomes are provided in the supplementary data set S1, Supplementary Material online.

### Evolutionary Analysis

Nucleotide and protein sequences were aligned with CLUSTALX (Thompson et al. 1997) and modified by eye with Bio-Edit (Hall 1999). The best-fit substitution models were selected using the jModelTest2 program for each data set according to the Akaike information criterion and the Bayesian information criterion (Darriba et al. 2012). Maximum likelihood (ML) and Bayesian phylogenetic trees were reconstructed with PhyML version 3.1 (Guindon et al. 2010) and MrBayes version 3.1.2 (Huelsenbeck and Ronquist 2001), respectively, following a previous study (Lin et al. 2014). PhyML 3.1 used the nearest neighbor interchange algorithm to search ML trees with 100 bootstrap replicates, whereas MrBayes 3.1.2 ran 1 million generations to reach the convergence with the standard deviation (SD) of split frequencies lower than 0.01. The ancestral sequences of whales were reconstructed using the Bayesian approach implemented in the BASEML program from the PAML package (Yang 2007). Nonsynonymous (*d*_N_) and synonymous (*d*_S_) distances and their standard errors from between sequences were estimated using the modified Nei–Gojobori method (Zhang et al. 1998) implemented in MEGA5 (Tamura et al. 2011). The ML estimates of the ratios of nonsynonymous and synonymous nucleotide substitution rates (ω = *d*_N_/*d*_S_) were generated by the CODEML program in PAML (Yang 2007).

## Results

### Evolution of Taste Receptor Genes in Whales

To determine how widespread the taste loss is in cetaceans, we examined all taste receptor genes that are responsible for five primary taste modalities in mammals: Sweet, umami, bitter, sour, and salty. First, we sequenced all members of the sweet/umami (3 *T1R*s) and bitter (10 *T2R*s) receptor gene families in six toothed whales and five baleen whales, and we also obtained these genes in a fully sequenced genome of one additional toothed whale (*Lipotes vexillifer*). Second, we analyzed three whole genome sequences of two toothed whales (*T. truncates* and *Li. vexillifer*) and one baleen whale (*B. acutorostrata*) to survey the sour taste marker gene (*Pkd2l1*) and the candidate salty taste receptor genes (*Scnn1a, Scnn1b,* and *Scnn1g*).

#### Pseudogenization of Sweet/Umami (T1Rs) and Bitter (T2Rs) Taste Receptor Genes

Mammals typically have only three members of *T1R* genes (*T1R1*, *T1R2*, and *T1R3*), among which *T1R1* and *T1R3* encode two subunits of the umami taste receptor, whereas *T1R2* and *T1R3* encode two proteins that function as the sweet taste receptor (Nelson et al. 2001, 2002; Sainz et al. 2001; Li et al. 2002; Zhao et al. 2003). *T1R*s include six exons: The first five encode the extracellular domain, whereas the sixth exon encodes all remaining domains of the receptors (Li et al. 2002). We successfully amplified *T1R1* (exon 6), *T1R2* (exons 3 and 6), and *T1R3* (exon 3) from six toothed whales and five baleen whales. All newly obtained sequences were characterized by frame-shifting indels (deletions or insertions) and/or premature stop codons, which are hallmarks of pseudogenes (fig. 1 and supplementary table S2, Supplementary Material online). This finding suggests that all *T1R*s in the examined whales are pseudogenes. Moreover, we identified one 1-bp deletion of *T1R3* and one premature stop codon of *T1R1* that are shared between toothed and baleen whales, which are indicative of an ancestral pseudogenization in the common ancestor of whales. However, we failed to identify any common disruptive mutations from both exons 3 and 6 of *T1R2* (fig. 1 and supplementary table S2, Supplementary Material online). Because *T1R3* encodes the shared subunit of the sweet and umami taste receptors, our results strongly suggest that sweet and umami tastes were lost in the common ancestor of whales.

Bitter taste receptors are encoded by *T2R*s, which lack introns and are ∼900 bp in length (Adler et al. 2000; Chandrashekar et al. 2000). We attempted to sequence ten *T2R*s that are orthologous to those identified from the dolphin genome (Jiang et al. 2012). With one exception, all amplified *T2R*s have disrupted open reading frames (ORFs) characterized by frame-shifting indels and/or premature stop codons (fig. 1 and supplementary table S2, Supplementary Material online). The exception is *T2R16*, which is intact in the three baleen whales (fig. 1). We also computed the nonsynonymous (*d*_N_) to synonymous (*d*_S_) distances for *T2R16* using MEGA5 (Tamura et al. 2011); the *d*_N_/*d*_S_ ratio does not significantly differ from 1 in all pairwise comparisons of the three species (mean ± SD, 0.48 ± 0.07; *P* > 0.1, *Z* test). In support of this finding, we estimated the mean *d*_N_/*d*_S_ ratio of *T2R16* pseudogenes (mean ± SD, 0.70 ± 0.35) and that of other *T2R* pseudogenes in baleen whales (mean ± SD, 1.37 ± 0.78), both mean *d*_N_/*d*_S_ ratios are not significantly different from 0.48, the mean *d*_N_/*d*_S_ ratio of *T2R16* intact genes (*P* > 0.05, two-tailed *t*-test). Thus, *T2R16* appears to undergo relaxation of functional constraints in the three baleen whales, which is comparable to the pseudogenizations in two additional baleen whales and five toothed whales (fig. 1). Among the remaining nine *T2R* pseudogenes, eight contain shared frame-shifting indels and/or premature stop codons between toothed and baleen whales, whereas one (*T2R5*) includes these pseudogene hallmarks shared in toothed whales only. The pseudogenization of *T2R5* may have occurred in baleen whales earlier than toothed whales because we failed to obtain this gene from PCR amplifications for five baleen whales and from the mink whale genome (fig. 1 and supplementary fig. S1*A*, Supplementary Material online). Indeed, although *T2R5* is flanked with *PRSS37* and *T2R3* in the bottlenose dolphin and Yangtze River dolphin genomes, we found that the two genes are adjacent to each other in scaffold_20 of the minke whale genome, suggesting a true loss of *T2R5* in the minke whale. In addition, we identified comparable numbers of *T2R*s in the three whale genomes (10 in bottlenose dolphin, 11 in Yangtze River dolphin, and 13 in mink whale), and we were able to find all ten *T2R*s in the Yangtze River dolphin and nine of ten *T2R*s in the mink whale (supplementary fig. S1*A*, Supplementary Material online), suggesting that the ten *T2R*s could represent the bitter taste receptor gene repertoire in all whales. All *T2R*s identified from the three whale genomes are pseudogenes, except the intact *T2R16* in the minke whale. Together, these results strongly indicate that bitter taste has been lost in all whales examined, and the relaxation of selective constraint on bitter taste receptor genes most likely occurred in the common ancestor of whales.

Given that toothed and baleen whales diverged 36 Ma and whales and even-toed ungulates diverged 53 Ma (Hasegawa et al. 2003; McGowen et al. 2009), our genetic evidence suggests that the pseudogenizations of sweet, umami, and bitter taste receptors took place in the common ancestor of whales between 36 and 53 Ma (fig. 1), probably shortly after 53 Ma, because we observed a number of common frame-shifting mutations in many genes predating the divergence of both major lineages of whales (fig. 1 and supplementary table S2, Supplementary Material online).

#### Pseudogenization of Sour Taste Marker Gene (Pkd2l1)

**Fig. 2.—:**
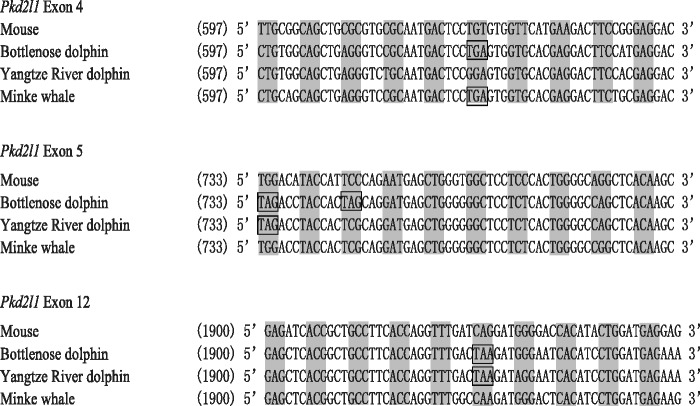
An alignment of *Pkd2l1* containing the ORF-disrupting mutations in two toothed whales (bottlenose dolphin and Yangtze River dolphin), one baleen whale (mink whale), and the outgroup (mouse). Codons in the correct reading frame are indicated by shading, and premature stop codons are boxed; the numbers in parentheses indicate the nucleotide positions following the mouse sequence.

Two sour taste marker genes (*Pkd1l3* and *Pkd2l1*), the expression of which is indispensable in sour taste functioning, encode the transient receptor potential channel members that were proposed to function as candidate sour taste receptors (Huang et al. 2006; Ishimaru et al. 2006). We used mouse genes as a query to search against the draft genome sequences of the dolphin, Yangtze River dolphin, and minke whale (Zhou et al. 2013; Yim et al. 2014). We failed to identify unambiguous sequences of *Pkd1l3* due to incomplete sequencing but instead obtained nearly complete coding sequences of *Pkd2l1* (supplementary table S2, Supplementary Material online). After aligning the three newly identified sequences with mouse *Pkd2l1* (GenBank accession no. NM_181422), we found multiple premature stop codons in each of the three whales with available genome sequences (fig. 2 and supplementary table S2, Supplementary Material online), which suggested that none of these sequences are functional. In the dolphin and minke whale sequences, the first premature stop codon is located at exon 4, leading to the loss of functional domains from exon 5 to exon 15. In the sequence of the Yangtze River dolphin, the 5′-most premature stop codon resides at exon 5, rendering the remaining ten exons nonfunctional. Furthermore, the dolphin and minke whale share the first premature stop codon located at exon 4, which indicates the possible pseudogenization of *Pkd2l1* in the common ancestor of whales, although exon 4 of the Yangtze River dolphin is complete and intact (fig. 2 and supplementary table S2, Supplementary Material online). In addition, two common premature stop codons were found in the dolphin and Yangtze River dolphin (fig. 2 and supplementary table S2, Supplementary Material online), suggesting a loss of function for *Pkd2l1* that predates the divergence of the two whales after separation from the minke whale. To determine when the functional relaxation of *Pkd2l1* occurred, we also inferred the *Pkd2l1* sequence of the common ancestor of the three whales (blue circle in supplementary fig. S1*B*, Supplementary Material online) using a Bayesian approach (Yang et al. 1995) and estimated the ω ratios of *Pkd2l1* for the common ancestor and 16 nonwhale mammals (supplementary fig. S1*B*, Supplementary Material online). We found that a model that allows a variation in ω between the common ancestor (ω_1_ = 0.15) and all 16 nonwhale mammals (ω_2_ = 0.35) is significantly better than a simpler model that assumes the same ω (ω_0_ = 0.16) across the tree (*P* = 0.016), which suggested that a functional relaxation of *Pkd2l1* arose in the common ancestor of whales.

#### Purifying Selection on Candidate Salty Taste Receptor Genes (Scnn1a, Scnn1b, and Scnn1g)

The epithelial sodium channel ENaC has been proposed to function as a candidate salty taste receptor, which usually involves three subunits encoded by *Scnn1a, Scnn1b,* and *Scnn1g* (Heck et al. 1984; Canessa et al. 1994; Firsov et al. 1998; Yarmolinsky et al. 2009). We examined the draft genome sequences of the dolphin, Yangtze River dolphin, and minke whale. In contrast to the widespread pseudogenization of other taste receptor genes, we were able to identify three salty taste receptor genes with nearly complete coding regions and intact ORFs (supplementary table S2, Supplementary Material online), which suggested that salty taste is retained in the three whales. Consistent with this finding, the *d*_N_/*d*_S_ ratio of each of the three genes was significantly lower than 1 in all pairwise comparisons of the three species (*P* < 0.001, *Z* test). These results indicate that the candidate salty taste receptor genes have experienced strong purifying selection, and salty taste may be functional in whales.

### Relaxation of Selective Constraint on Taste Signaling Pathways in Whales

To test whether widespread losses of taste receptor genes have affected the evolution of taste signaling pathways, we examined three genes (*Calhm1, Trpm5,* and *Plcβ2*) that are known to be involved in taste signaling (Zhang et al. 2003; Taruno et al. 2013). Specifically, *Calhm1* encodes the CALHM1 ion channel that is required for the neurotransmission of sweet, bitter, and umami tastes (Taruno et al. 2013); *Trpm5* and *Plcβ2* encode a TRP ion channel and a phospholipase C, respectively, both of which are common signaling molecules for sweet, umami, and bitter taste transduction (Zhang et al. 2003).

**Fig. 3.—:**
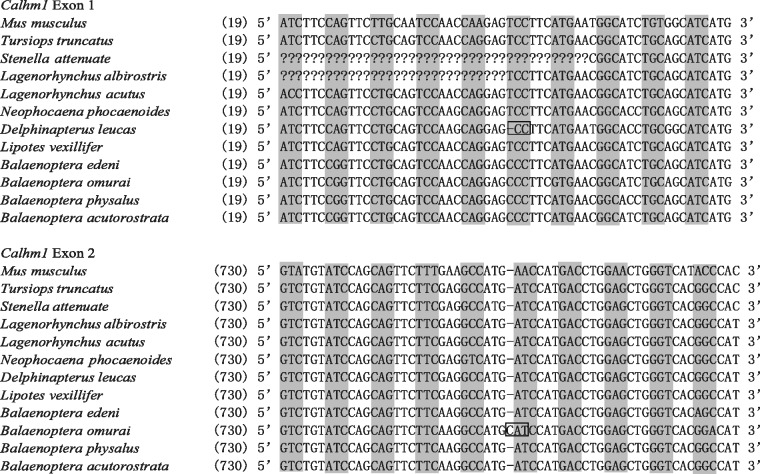
An alignment of *Calhm1* containing the ORF-disrupting mutations in seven toothed whales and four baleen whales. Dashes indicate alignment gaps, and question marks represent unamplified nucleotides. Codons in the correct reading frame are indicated by shading, whereas codons containing one nucleotide deletion or insertion are boxed.

**Fig. 4.—:**
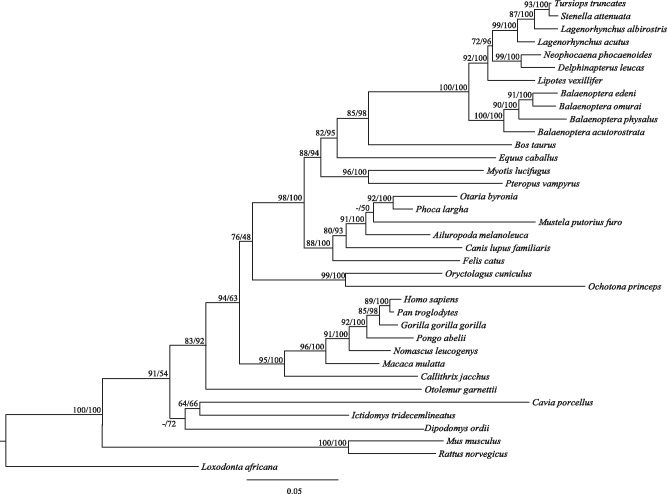
The ML tree of *Calhm1* in mammals under the GTR+I+G substitution model of sequence evolution. Branch lengths are drawn to scale. ML bootstrap values/Bayesian posterior probabilities (>50%) are shown as numbers above the branches.

We sequenced all two coding exons of *Calhm1* from six toothed whales and four baleen whales and identified an additional *Calhm1* from the genome of one toothed whale (Yangtze River dolphin) (figs. 3 and 4). With two exceptions, the ORF of *Calhm1* is intact in nine other sequenced whales, suggesting a functional role in most whales. The first exception is one toothed whale (*Delphinapterus leucas*), which showed one 1-bp deletion in exon 1, whereas the other exception is a baleen whale (*B. omurai*), where one 1-bp insertion in exon 2 occurred (fig. 3). These indels result in a shift in the ORF and could create multiple premature stop codons, which are indicative of a loss of function. Moreover, the lack of common ORF-disrupting indels in the two whales suggests independent events of the relaxation of functional constraint. To determine whether the relaxation of functional constraint occurred before or after the radiation of extant whales, we estimated the ω ratios of *Calhm1* in whales using a likelihood approach. We computed likelihood tests on two data sets. Data set I included 26 nonwhale mammals and one ancestral sequence of all whales, whereas data set II contained all mammals (26 nonwhale mammals and 11 whales) after the removal of premature stop codons (table 1). First, we examined data set I under the assumption of a uniform ω across the tree (model A in table 1); ω was estimated to be 0.06, which suggested an overall purifying selection on *Calhm1* in mammals. Assuming that the ancestral sequence of all whales has ω_2_ and other branches have ω_1_ (model B in table 1), we estimated ω_2_ = 0.17, and model B fits the data significantly better than model A (*P* = 0.021), indicating the common ancestor of all whales has a significantly higher ω than other mammals. This result supports the hypothesis stating that a relaxation of functional constraint started from the common ancestor of all whales. Indeed, the functional relaxation of *Calhm1* is well reflected by the elevated branch length of the ancestral branch leading to whales (fig. 4). Second, we analyzed data set II and allowed a variation in ω between the stem whale branch and all branches connecting 11 whales (model D in table 1). The stem whale branch was estimated to have a significantly lower ω than the other whale branches (*P* = 0.01) after comparing model D with model C, the latter of which is a simpler model assuming the same ω for all whale branches (table 1). This analysis suggests the further relaxation of functional constraint after the divergence of whales. In addition, we found that a model allowing a variation in ω between toothed whales and baleen whales is significantly better than a simpler model estimating the same ω for both major lineages of whales (*P* = 0.04, χ^2^ test). This result suggests differential levels of selective pressure acting on toothed and baleen whales.

**Table 1:** Likelihood Ratio Tests of Selective Pressures on *Calhm1, Trpm5, and Plcβ2* in Mammals

Models	ω (*d*_N_/*d*_S_)	Comparisons	*P*
Data set I: 27 *Calhm1* sequences (26 nonwhale mammals plus the ancestral sequence of all whales)			
A. All branches have the same ω	ω = 0.06		
B. Ancestral branch of all whales has ω_2_ and other branches have ω_1_	ω_1_ = 0.06, ω_2_ = 0.17	B vs. A	**0.021**
Data set II: 37 *Calhm1* sequences (26 nonwhale mammals plus 11 whales)			
C. Ancestral branch of all whales and branches connecting 11 whales have ω_2_, whereas other branches have ω_1_	ω_1_ = 0.06, ω_2_ = 0.60		
D. Ancestral branch of all whales has ω_3,_ branches connecting 11 whales have ω_2_, and other branches have ω_1_	ω_1_ = 0.06, ω_2_ = 0.69, ω_3_ = 0.16	D vs. C	**0.010**
Data set III: 15 *Trpm5* sequences (14 nonwhale mammals plus the ancestral sequence of all whales)			
E. All branches have the same ω	ω = 0.09		
F. Ancestral branch of all whales has ω_2,_ and other branches have ω_1_	ω_1_ = 0.09, ω_2_ = 0.15	F vs. E	0.203
Data set IV: 17 *Trpm5* sequences (14 nonwhale mammals plus 3 whales)			
G. Ancestral branch of all whales and branches connecting 3 whales have ω_2,_ whereas other branches have ω_1_	ω_1_ = 0.09, ω_2_ = 0.37		
H. Ancestral branch of all whales has ω_3,_ branches connecting three whales have ω_2_, and other branches have ω_1_	ω_1_ = 0.09, ω_2_ = 0.44, ω_3_ = 0.12	H vs. G	**0.005**
Data set V: 14 *Plcβ2* sequences (13 nonwhale mammals plus the ancestral sequence of all whales)			
I. All branches have the same ω	ω = 0.11		
J. Ancestral branch of all whales has ω_2,_ and other branches have ω_1_	ω_1_ = 0.11, ω_2_ = 0.25	J vs. I	**0.007**
Data set VI: 16 *Plcβ2* sequences (13 nonwhale mammals plus 3 whales)			
K. Ancestral branch of all whales and branches connecting three whales have ω_2,_ whereas other branches have ω_1_	ω_1_ = 0.11, ω_2_ = 0.19		
L. Ancestral branch of all whales has ω_3,_ branches connecting three whales have ω_2_, and other branches have ω_1_	ω_1_ = 0.12, ω_2_ = 0.16, ω_3_ = 0.25	L vs. K	0.254

Note.—Significant *P* values (< 0.05) are indicated in bold.

**Fig. 5.—:**
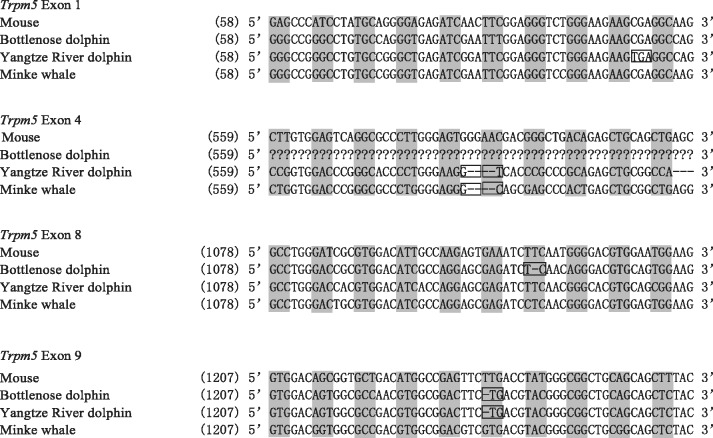
An alignment of *Trpm5* containing the ORF-disrupting mutations in two toothed whales (bottlenose dolphin and Yangtze River dolphin), one baleen whale (mink whale), and the outgroup (mouse).

We attempted to identify all 24 coding exons of *Trpm5* and all 32 coding exons of *Plcβ2* in the three whales with available genome sequences (fig. 5, supplementary table S2, Supplementary Material online). In the three *Trpm5* sequences, we detected multiple premature stop codons and/or frame-shifting mutations, which are indicative of a *Trpm5* pseudogene in each of the three whales (supplementary table S2, Supplementary Material online). Notably, a 4-bp ORF-disruptive deletion in exon 4 was shared between a toothed whale (Yangtze River dolphin) and a baleen whale (minke whale), suggesting the possibility of pseudogenization of *Trpm5* in the common ancestor of whales. Additionally, we observed a 1-bp ORF-disruptive deletion in exon 9 shared in the two toothed whales (bottlenose dolphin and Yangtze River dolphin). In contrast to *Trpm5*, *Plcβ2* was found to be complete and intact in the three whales (supplementary table S2, Supplementary Material online), which indicated a functional role. To determine whether the relaxation of functional constraint occurred along the ancestral lineage of whales, we conducted a series of selection tests on *Trpm5* and *Plcβ2* that are similar to those on *Calhm1* (table 1) using phylogenetic trees as shown in supplementary fig. S1*C* (*Trpm5*) and *D* (*Plcβ2*), Supplementary Material online. First, we analyzed data set III, which consisted of 14 nonwhale mammals and the ancestral sequence of all whales. We found that model F, which allowed a variation in ω between the ancestral branch and other branches, is not significantly better than model E, which features a uniform ω across the tree (*P* = 0.203). However, under the assumption of differential ω ratios between the ancestral branch leading to whales and the four branches connecting the three whales (model H), we found that model H to fit data set IV significantly better than model G, which assumes no ω variation for these five branches (*P* = 0.005). Hence, these findings suggest that the relaxation of the functional constraint of *Trpm5* did not originate in the ancestral branch of whales but may instead have occurred after the divergence of whales. Second, we examined data set V of 14 *Plcβ2* sequences and found the ancestral branch of all whales to have a significantly higher ω than other mammals (*P* = 0.007) after comparing model J with model I (table 1). Nevertheless, we did not find model L to be significantly better than model K (*P* = 0.26), which indicates similar levels of selective pressure on the *Plcβ2* between the ancestral branch and branches connecting the three examined whales. Therefore, the relaxation of the functional constraint on *Plcβ2* may have occurred in the common ancestor of whales, although we failed to identify ORF-disruptive mutations in the sequences. In addition, we did not find significant differences in ω between toothed and baleen whales for *Trpm5* and *Plcβ2* (*P* > 0.4).

Collectively, our evolutionary analyses suggest that the relaxation of functional constraints on taste signal transduction likely took place in the common ancestor of whales between 36 and 53 Ma, possibly shortly before 36 Ma, because the relaxed selection is weak, as indicated by small ω ratios along the ancestral branch leading to whales (table 1). Thus, the relaxation of functional constraints on taste signal transduction must have occurred after the pseudogenizations of taste receptor genes, probably because of other functions involved in these transduction pathways (Damak et al. 2006; Taruno et al. 2013). In addition, this relaxation is not a part of any genome-wide features, because the molecular evolutionary rate has generally slowed down in whales (McGowen et al. 2012; Zhou et al. 2013; Yim et al. 2014).

## Discussion

In this study, we surveyed all five primary taste receptor genes in toothed and baleen whales to examine the generality of whale taste loss and test whether taste loss has influenced the evolution of taste signal transduction. With the exception of the intact *T2R16* in the three baleen whales, we found all members of sweet/umami (3 *T1R*s) and bitter (10 *T2R*s) receptor gene families (if amplified) to be pseudogenes in all 12 examined whales, with a shared premature stop codon in 10 of 13 genes, demonstrating massive losses of taste receptor genes in the common ancestor of toothed and baleen whales. Furthermore, we found that the sour taste marker gene *Pkd2l1* is a pseudogene, whereas the candidate salty taste receptor genes (*Scnn1a, Scnn1b,* and *Scnn1g*) are intact and evolutionarily conserved in two toothed whales and one baleen whale with available genome sequences. Finally, we showed the relaxation of selective constraints on taste signaling pathways in the common ancestor of whales (*Calhm1* and *Plcβ2*) or after the divergence of whales (*Trpm5*). Our findings unambiguously suggest widespread losses of umami, sweet, bitter, and sour tastes and the evolutionary conservation of salty taste in whales. The major reduction of taste receptor genes in whales may have resulted in the relaxation of selective constraints on taste signaling pathways. Earlier studies discovered the loss of one or two primary tastes in certain groups of animals; our genetic study suggests that whales represent the first group of animals that strikingly lack four of five primary tastes.

Consistent with our genetic evidence, anatomical studies have revealed taste buds in small pits at the base of dolphin tongues and few taste bud-like structures in the canonical taste structures (lingual papillae) of the four toothed whales (Yoshimura and Kobayashi 1997). Furthermore, taste buds were not discovered in seven other toothed whales (Arvy and Pilleri 1970; Yamasaki et al. 1976; Kuznetzov 1990; Pfeiffer et al. 2001). Behavioral tests of whale taste sensation are scant, with a few studies having examined bottlenose dolphins (Nachtigall and Hall 1984; Friedl et al. 1990). The dolphins could detect sweet, bitter, sour, and salty tastants with an order of magnitude below human sensitivities (Friedl et al. 1990) or show indifference or reduced sensitivity to sweet and bitter tastants (Kuznetsov 1974). These anatomical and behavioral examinations could have been biased because of small sample sizes and few comparisons of sex and age differences. However, the morphological characteristics of the whale tongues are generally simplified, and their taste sensitivity is poorly developed.

Why could whales afford to lose four of five primary tastes? We provide three probable explanations. First, the feeding behavior of swallowing prey whole without mastication may have rendered their tastes useless, as suggested by two recent studies (Jiang et al. 2012; Sato and Wolsan 2012). Second, their food items are likely to possess reduced taste stimuli because a high concentration of sodium in the ocean could have masked the tastant cues, which could be quickly diluted in sea water (Ikeda 1909; Komata 1990). Third, a dietary switch from plants to meat in the whale ancestor may account for the major loss of sweet and bitter tastes, because meat contains little sweet and bitter compounds. This scenario is analogous to that occurred in the giant panda, which has lost its umami taste due to a dietary change from meat to bamboo (Zhao, Yang, et al. 2010). The extinct even-toed ungulates (Raoellidae) that are most closely related to whales were plant eaters, which strongly suggests that a major dietary change occurred during the transition from even-toed ungulates to whales (Thewissen et al. 2007).

In contrast, the maintenance of the only primary taste, salty taste, is not unexpected. The sodium channel ENaC, which functions as the candidate salty taste receptor, typically consists of a multimeric complex of three subunits and plays a major role in the regulation of extracellular volume and blood pressure (Canessa et al. 1994). Each of the three subunits showed significant sequence similarity and similar expression patterns between invertebrates and mammals (Chang et al. 1996; Ottaviani et al. 2002). This evolutionary conservation of ENaC strongly suggests its functional indispensability across animals. Indeed, the hereditary mutations of ENaC subunits were demonstrated to result in Liddle’s syndrome of hypertension (Schild et al. 1996); disruptive mutations creating dysfunction of ENaC were found to cause pseudohypoaldosteronism with severe sodium loss (Chang et al. 1996; Kerem et al. 1999). As sea-living organisms, whales are well adapted to their hyperosmotic environment; osmoregulation is required to maintain the ENaC function that regulates sodium ion fluxes and thus retains the salty taste (Ottaviani et al. 2002).

Taste receptor evolution in relation to feeding ecology is not well understood. The evolutionary patterns of taste receptor genes matched the variations of feeding ecology in the giant panda (Zhao, Yang, et al. 2010), humans (Wang et al. 2004), vampire bats (Zhao et al. 2010), carnivores (Jiang et al. 2012), and vertebrates in general (Li and Zhang 2014), but mismatches between taste receptor evolution and feeding ecology were also observed (Zhao and Zhang 2012; Zhao et al. 2012). Thorough scrutiny of the physiological functions of taste receptor genes should help to understand the connection between taste function and feeding ecology. Indeed, in addition to a gustatory system, taste receptors are also involved in extragustatory systems, such as the lung, gut, brain, and testis (Behrens and Meyerhof 2011), which suggests that these extragustatory functions may account for the aforementioned mismatches. Nonetheless, the massive losses of taste receptor genes in whales are striking, although losses of taste receptors have been frequently identified from insects to mammals (Nei et al. 2008; Xiao et al. 2013). Therefore, taste receptors that are pseudogenized could not be involved in extraoral functions in whales; the functional significance of these extraoral taste receptors in humans and rodents must be unimportant in these marine mammals. Future studies of other sensory systems that are still maintained in whales may provide a better understanding of how whales and other marine animals can sense and survive in the ocean.

## Supplementary Material

Supplementary figure S1, tables S1 and S2, and data set S1 are available at *Genome Biology and Evolution* online (http://www.gbe.oxfordjournals.org/).

Supplementary Data
