# Supplementary material for: Massive Losses of Taste Receptor Genes in Toothed and Baleen Whales
Source: Genome Biol Evol. 2014 May 6;6(6):1254–65. doi: 10.1093/gbe/evu095 (PMC4079202; doi:10.1093/gbe/evu095)
Supplement: Supplementary Data [file supp_6_6_1254__index.html]

Massive losses of taste receptor genes in toothed and baleen whales — Massive Losses of Taste Receptor Genes in Toothed and Baleen Whales — Supplementary Data 

# Massive Losses of Taste Receptor Genes in Toothed and Baleen Whales

## Supplementary Data

files

**Files in this Data Supplement:**

- Supplementary Data - zip file
