# Supplementary material for: Massive Losses of Taste Receptor Genes in Toothed and Baleen Whales
Source: Genome Biol Evol. 2014 May 6;6(6):1254–65. doi: 10.1093/gbe/evu095 (PMC4079202; doi:10.1093/gbe/evu095)
Supplement: Supplementary Data [file supp_evu095_suppl_data.zip › Table_S1-S2.docx]

**Table S1.** Primer sequences in this study.

| Primer pair | Primer sequence^1^ | Ta^2^ (^o^C) | Gene fragment | Target length |
| --- | --- | --- | --- | --- |
| 1011 | cttcccttccagACCCCTAC | 50-65 ^o^C | *T1R1* Exon 6 | 932bp |
| 1012 | ggTCAGGTGGAGCCACAG |  |  |  |
|  |  |  |  |  |
| 1017 | CCTCCGGCAGATCACC | 50-65 ^o^C | *T1R2* Exon 3 | 774bp |
| 1018 | cagacctcacCTGCCAAGG |  |  |  |
|  |  |  |  |  |
| 1021 | tgggctcttgcagGTGAAT | 50-65 ^o^C | *T1R2* Exon 6 | 929bp |
| 1022 | CTATCCCCTCCCCATGGTGTAGCC |  |  |  |
|  |  |  |  |  |
| 1025 | ggacccgcagGTCAGCTA | 50-65 ^o^C | *T1R3* Exon 3 | 783bp |
| 1026_1 | ccgtgccctcacCTG |  |  |  |
| 1026_2 | AGAGCAGCGTGTTGTGGAG |  |  |  |
|  |  |  |  |  |
| 1057 | TCATTAGCCACCTTTGTTTGG | 50-65 ^o^C | *T2R1* | 868bp |
| 1058 | GCACTTTCTGTGGAGGAGCA |  |  |  |
|  |  |  |  |  |
| 1059 | GGCGATTTCCAGGATTTGTC | 50-65 ^o^C | *T2R1* | 732bp |
| 1060 | GCACTTTCTGTGGAGGAGCA |  |  |  |
|  |  |  |  |  |
| 1047 | GGCCTCCTCTTTGTCAGCTC | 50-65 ^o^C | *T2R2* | 897 bp |
| 1048 | TTTCTGAGGTAGAGAAGTCTCCTG |  |  |  |
|  |  |  |  |  |
| 1049 | TCCTCTTTGTCAGCTCGTC | 50-65 ^o^C | *T2R2* | 808 bp |
| 1050 | TGCTGCCATTATGTCCTTC |  |  |  |
|  |  |  |  |  |
| 1053 | CTGCTGTGGATTCTCTTGATTG | 50-65 ^o^C | *T2R3* | 770bp |
| 1054 | CTATGGAAAAACGGGTTCCT |  |  |  |
|  |  |  |  |  |
| 1055 | CCAAACTCCACGATGAATAA | 50-65 ^o^C | *T2R3* | 711bp |
| 1056 | TCCAGGCTTCAGATGACCAG |  |  |  |
|  |  |  |  |  |
| 1051 | CCTGGTTGGAAATGGAGTC | 50-65 ^o^C | *T2R5* | 681bp |
| 1052 | AAAGACCTGGAGGTGATGG |  |  |  |
|  |  |  |  |  |
| 1061 | TGATAACCATCCAACTCTGTCTTC | 50-65 ^o^C | *T2R16* | 890bp |
| 1062 | AGCCTCTAGGTCCCAGCAC |  |  |  |
|  |  |  |  |  |
| 1063 | TGGGCACAGAGTGGGTAAGT | 50-65 ^o^C | *T2R16* | 741bp |
| 1064 | ATCCGTGAAGTCAAATGAATAGAG |  |  |  |
|  |  |  |  |  |
| 1065 | TTGCAGTAGGGATCCTGGTC | 50-65 ^o^C | *T2R38* | 883bp |
| 1066 | GCCCTTACCTTTAGGCTGCT |  |  |  |
|  |  |  |  |  |
| 1033 | ATGACTGAAACCTGCAATCC | 50-65 ^o^C | *T2R39* | 958bp |
| 1034 | TAAAGGTGAACTCCGCTTCC |  |  |  |
|  |  |  |  |  |
| 1035 | AAATGGGTTCATTGTGGCT | 50-65 ^o^C | *T2R39* | 805bp |
| 1036 | CAGGGTTGTCCTGTATCAGTAGG |  |  |  |
|  |  |  |  |  |
| 1037 | TGGTGGCAGTGGTGGGTA | 50-65 ^o^C | *T2R60* | 808bp |
| 1038 | TCTCGGTCTGCTGTTACTC |  |  |  |
|  |  |  |  |  |
| 1039 | GAGTGGAGAGGACGTGGTTC | 50-65 ^o^C | *T2R60* | 896bp |
| 1040 | ACAGCTCTCGGTCTGCTGTT |  |  |  |
|  |  |  |  |  |
| 1041 | CAGGACCTCAGTTGGCTGAT | 50-65 ^o^C | *T2R60* | 816bp |
| 1042 | TAAATCACAGCCTGCCACAC |  |  |  |
|  |  |  |  |  |
| 1043 | CCCTCCTCACCCATGTTG | 50-65 ^o^C | *T2R62*a | 781bp |
| 1044 | CAGCGCCAGTGATTCCAG |  |  |  |
|  |  |  |  |  |
| 1045 | GTGGGTACAATGCCAGAC | 50-65 ^o^C | *T2R62*a | 677 bp |
| 1046 | CAGTGATTCCAGAGGGTTA |  |  |  |
|  |  |  |  |  |
| 1067 | CAGAGCGGCTCATCCA | 50-65 ^o^C | *T2R62*b | 648bp |
| 1068 | CGGTGCAGCGAGAACA |  |  |  |
|  |  |  |  |  |
| 1069 | AGAGCCTCTGGGCCTGTAGT | 50-65 ^o^C | *T2R62*b | 810bp |
| 1070 | TGGGATGTTCACAAGGACAA |  |  |  |
|  |  |  |  |  |
| 1133 | GGCACCATGGACAAGTTCCG | 50-65 ^o^C | *Calhm1* Exon 1 | 555bp |
| 1134 | GCCATCTGGCCCCTTAGCTG |  |  |  |
|  |  |  |  |  |
| 1135 | GGCACCATGGACAAGT | 50-65 ^o^C | *Calhm1* Exon 1 | 555bp |
| 1136 | GAGATGCAGTGCAGGTAG |  |  |  |
|  |  |  |  |  |
| 1137 | GTCACTGGGCTGGTCCTT | 50-65 ^o^C | *Calhm1* Exon 2 | 483bp |
| 1138 | CATTCCTGCCTCTTCACC |  |  |  |
|  |  |  |  |  |
| 1141 | CGTGCTGCTGACCACA | 50-65 ^o^C | *Calhm1* Exon 2 | 483bp |
| 1142 | TGCTGAAGTAGGTGGC |  |  |  |
|  |  |  |  |  |
| 1143 | GTTCGTCGTGCGCTCTG | 50-65 ^o^C | *Calhm1* Exon 2 | 483bp |
| 1144 | TTGCTGAAGTAGGTGGC |  |  |  |

^1^Locations of nucleotides are indicated in lowcase (in introns) or uppercase (in exons).

^2^Touchdown PCR program was used with annealing temperatures from 65^o^C to 50^o^C.

**Table S2.** Frame-shifting mutations of taste receptor genes. (A) *T1R*s and *T2R*s; (B) *Pkd2l1*; (C) *Scnn1a*, *Scnn1b and Scnn1g*; (D) *Trpm5*; (E) *Plcβ2.* Insertions (↓), deletions (↑), and premature stop codons (*) are shown, the shared ones across whales are indicated in red. Sizes of indels are given in base pairs. “NA” means the sequence information is not available.

**(A)**

| Species | *T1R1* (Ex6) | *T1R2* (Ex3) | *T1R2* (Ex6) | *T1R3* (Ex3) | *T2R1* | *T2R2* | *T2R3* |
| --- | --- | --- | --- | --- | --- | --- | --- |
| *Tursiops truncates* | 2↑,* | 15↓,5↓,6↓ | 1↑,1↑,3↑ | 1↑,3↑,1↑ | 3↑,3↓,1↑,3↓,* | 3↑,1↑,1↑,* | 1↑,1↑ |
| *Globicephala melas* | 0, * | 15↓,5↓,6↓ | 6↑,1↑,1↑,3↑ | 1↑ | NA | NA | 1↑,1↑,1↓ |
| *Lagenorhynchus albirostris* | 1↑,* | 15↓,5↓,6↓,2↑ | 1↑,6↑,1↑ | 1↑,1↑ | 3↑,3↓,1↑,3↓,*,4↑ | 3↑,1↑,1↑,*,2↓ | 4↑,4↑,1↑,1↑,1↓ |
| *L. acutus* | 3↑,1↑,* | 12↑,15↓,5↓,6↓,2↑ | 6↑,1↑,1↑,1↑ | NA | 3↑,3↓,1↑,3↓,* | 1↑,3↑,1↑,1↑,* | 4↑,1↑,1↑ |
| *Neophocaena phocaenoides* | NA | NA | NA | 1↑, 1↑ | NA | NA | 4↑,4↑,1↑ |
| *Lipotes vexillifer* | 4↑,*,2↓ | 12↑,1↑,5↓,6↓ | 1↓ | 1↓ | 3↑,3↑,3↓,1↑,3↓,* | 3↑,1↑,1↑,*,2↓ | 1↑,4↑,4↑,2↑,32↑,5↑,1↑ |
| *Physeter catodon* | 3↑,* | 12↑,1↓,1↑,5↓,6↓ | 13↑,2↓ | 3↑,1↑, 35↑ | 3↑,3↓,3↓,* | 1↑,1↑,*,2↓ | 4↑,1↓,1↑,1↑ |
| *Balaenoptera edeni* | NA | NA | NA | NA | 3↑,3↓,3↓,* | 1↑,*,2↓ | 4↑,1↑,4↑,1↑ |
| *B. omurai* | NA | NA | NA | 1↑ | NA | 1↑,1↑,1↑,*5↑,2↓,1↑ | 4↑,1↑,4↑,1↑ |
| *B. physalus* | *,54↑ | NA | 1↑ | NA | NA | NA | 4↑ |
| *B. acutorostrata* | 1↓,*,54↑ | 12↑,6↓ | 1↑,9↑,5↑ | NA | 3↑,3↓,3↓,* | NA | 1↑ |
| *Balaena mysticetus* | *,54↑ | NA | 1↑ | NA | 3↑,3↓,3↓,* | NA | 4↑ |
|  | ***T2R5*** | ***T2R16*** | ***T2R38*** | ***T2R39*** | ***T2R60*** | ***T2R62a*** | ***T2R62b*** |
| *Tursiops truncates* | *,0 | 3↑,1↑,4↑ | 3↓,5↑,1↓,2↑,3↑ | 4↓,*,1↓,3↓ | *,4↑,2↑,9↑ | 1↓,*,4↑,1↓,1↑ | 5↓,3↑,1↑,2↑,43↑ |
| *Globicephala melas* | NA | NA | NA | 4↓,*,3↓ | NA | NA | 2↑,5↓,3↑,1↑,2↑,1↓ |
| *Lagenorhynchus albirostris* | *,0 | 3↑,3↑,1↑,4↑ | 3↓,5↑,1↓,2↑ | 4↓,*,3↓ | 1↑,*,4↑,1↑ | *,3↓,4↑,1↑ | 5↓,3↑,1↑,2↑,1↓ |
| *L. acutus* | *,0 | 3↑,1↑,4↑ | 3↓,5↑,1↓,2↑ | 4↓,*,3↓,1↓ | *,4↑,1↑ | *,3↓,4↑,1↓,1↑ | 2↑,3↑,1↑,2↑,1↓,11↑ |
| *Neophocaena phocaenoides* | NA | NA | NA | NA | NA | NA | NA |
| *Lipotes vexillifer* | 1↓,*,1↑,2↓,3↓ | 2↑,8↑,3↑,1↑,4↑ | 3↓,1↓,2↑,2↑ | 3↓,*,4↓,4↑ | *,4↑,1↑,3↓ | *,3↓,4↑,1↓,1↑ | 5↓,17↓,3↑,15↑,1↑,2↑,8↑,2↑,11↑ |
| *Physeter catodon* | 0 | 1↑,1↑,3↑,2↑,1↑,4↑ | 3↓,5↑,3↑,1↓,2↑ | 4↓,*,3↓ | *,1↑ | NA | 2↓,5↓,3↑,1↓,1↑,1↑,2↑ |
| *Balaenoptera edeni* | NA | 3↑,2↑ | NA | NA | NA | NA | 5↓,3↑ |
| *B. omurai* | NA | 2↑ | NA | NA | NA | NA | NA |
| *B. physalus* | NA | 3↑ | NA | 4↓,2↓,*,3↓ | NA | NA | NA |
| *B. acutorostrata* | NA | 3↑ | 3↓,1↓,2↑,1↑ | 4↓,*,3↓ | *,0 | *,3↓,1↓ | 5↓,3↑ |
| *Balaena mysticetus* | NA | 3↑,3↑ | NA | NA | NA | NA | NA |

**(B)**

| Species | Ex1 | Ex2 | Ex 3 | Ex 4 | Ex 5 | Ex 6 | Ex 7 | Ex 8 | Ex 9 | Ex 10 | Ex 11 | Ex 12 | Ex 13 | Ex 14 | Ex 15 |
| --- | --- | --- | --- | --- | --- | --- | --- | --- | --- | --- | --- | --- | --- | --- | --- |
| *Tursiops truncatus* | 0 | 0 | 0 | *, 2↑ | * | 0 | 3↑ | 0 | 0 | 0 | 0 | *,0 | 0 | 0 | NA |
| *Lipotes vexillifer* | 0 | 0 | 0 | 0 | *, 1↓ | 0 | 3↑ | 0 | 6↑ | 0 | 0 | *,0 | 0 | 1↑ | NA |
| *Balaenoptera acutorostrata* | 0 | 0 | 0 | *, 0 | 0 | 0 | 0 | 0 | 0 | *,0 | 0 | 0 | 0 | *,0 | NA |

**(C)**

| Exon (*Scnn1a*) | 1 | 2 | 3 | 4 | 5 | 6 | 7 | 8 | 9 | 10 | 11 | 12 |
| --- | --- | --- | --- | --- | --- | --- | --- | --- | --- | --- | --- | --- |
| *Tursiops truncatus* | NA | 6↑ | 3↑ | 0 | 0 | 0 | 0 | 0 | 0 | NA | 0 | 1↑,1↑,6↑ |
| *Lipotes vexillifer* | 0 | 6↑ | 3↑ | 0 | 0 | 0 | 0 | 0 | 0 | NA | 0 | 6↑ |
| *Balaenoptera acutorostrata* | 0 | 0 | 0 | 0 | 0 | 0 | 0 | 0 | 0 | NA | 0 | 6↑ |
|  |  |  |  |  |  |  |  |  |  |  |  |  |
| Exon (*Scnn1b*) | **1** | **2** | **3** | **4** | **5** | **6** | **7** | **8** | **9** | **10** | **11** | **12** |
| *Tursiops truncatus* | 0 | 9↓ | 0 | 0 | 0 | 0 | NA | 0 | 0 | 0 | 0 | 0 |
| *Lipotes vexillifer* | 0 | 9↓ | 0 | 0 | 0 | 0 | 0 | 0 | 0 | 0 | 0 | 0 |
| *Balaenoptera acutorostrata* | 0 | 9↓ | 0 | 0 | 0 | 0 | 0 | 0 | 0 | 0 | 0 | 0 |
|  |  |  |  |  |  |  |  |  |  |  |  |  |
| Exon (*Scnn1g*) | **1** | **2** | **3** | **4** | **5** | **6** | **7** | **8** | **9** | **10** | **11** | **12** |
| *Tursiops truncatus* | 0 | 9↑ | 0 | 0 | 0 | 0 | 0 | 0 | 0 | 3↑ | 0 | 0 |
| *Lipotes vexillifer* | 0 | 9↑ | 0 | 0 | 0 | 0 | 0 | 0 | 0 | 3↑ | 0 | 0 |
| *Balaenoptera acutorostrata* | 0 | 9↑ | 0 | 0 | 0 | 0 | 0 | 0 | 0 | 3↑ | 0 | 0 |

**(D)**

| Species | Ex1 | Ex 2 | Ex 3 | Ex 4 | Ex 5 | Ex 6 | Ex 7 | Ex 8 | Ex 9 | Ex 10 | Ex 11 | Ex 12 |
| --- | --- | --- | --- | --- | --- | --- | --- | --- | --- | --- | --- | --- |
| *Tursiops truncatus* | 0 | 0 | 0 | 0 | NA | 0 | NA | 1↑ | 1↑,12↑ | 0 | 1↓ | 2↓ |
| *Lipotes vexillifer* | *,0 | *,0 | 8↑ | 1↑,4↑,6↑ | NA | 0 | NA | 0 | 1↑,12↑ | *,0 | 0 | 0 |
| *Balaenoptera acutorostrata* | 1↑ | 0 | 0 | 4↑ | 0 | 0 | 0 | 0 | 0 | 0 | 0 | 0 |
|  |  |  |  |  |  |  |  |  |  |  |  |  |
|  | Ex **13** | Ex **14** | Ex **15** | Ex **16** | Ex **17** | Ex **18** | Ex **19** | Ex **20** | Ex **21** | Ex **22** | Ex **23** | Ex **24** |
| *Tursiops truncatus* | 0 | NA | 1↑ | 2↑ | NA | NA | 15↑ | 0 | 0 | 3↑ | NA | NA |
| *Lipotes vexillifer* | 1↑ | NA | 0 | NA | NA | NA | 15↑ | 1↑ | 0 | 3↑ | NA | NA |
| *Balaenoptera acutorostrata* | 0 | NA | 0 | 1↑ | 0 | 0 | 15↑ | 0 | 0 | NA | NA | NA |

**(E)**

| Species | Ex1 | Ex 2 | Ex 3 | Ex 4 | Ex 5 | Ex 6 | Ex 7 | Ex 8 | Ex 9 | Ex 10 | Ex 11 | Ex 12 |
| --- | --- | --- | --- | --- | --- | --- | --- | --- | --- | --- | --- | --- |
| *Tursiops truncatus* | 0 | 0 | 0 | 0 | NA | 0 | NA | 1↑ | 1↑,12↑ | 0 | 1↓ | 2↓ |
| *Lipotes vexillifer* | *,0 | *,0 | 8↑ | 1↑,4↑,6↑ | NA | 0 | NA | 0 | 1↑,12↑ | *,0 | 0 | 0 |
| *Balaenoptera acutorostrata* | 1↑ | 0 | 0 | 4↑ | 0 | 0 | 0 | 0 | 0 | 0 | 0 | 0 |
|  |  |  |  |  |  |  |  |  |  |  |  |  |
|  | Ex **13** | Ex **14** | Ex **15** | Ex **16** | Ex **17** | Ex **18** | Ex **19** | Ex **20** | Ex **21** | Ex **22** | Ex **23** | Ex **24** |
| *Tursiops truncatus* | 0 | NA | 1↑ | 2↑ | NA | NA | 15↑ | 0 | 0 | 3↑ | NA | NA |
| *Lipotes vexillifer* | 1↑ | NA | 0 | NA | NA | NA | 15↑ | 1↑ | 0 | 3↑ | NA | NA |
| *Balaenoptera acutorostrata* | 0 | NA | 0 | 1↑ | 0 | 0 | 15↑ | 0 | 0 | NA | NA | NA |
