# Supplementary figures and images for: Massive Losses of Taste Receptor Genes in Toothed and Baleen Whales
Source: Genome Biol Evol. 2014 May 6;6(6):1254–65. doi: 10.1093/gbe/evu095 (PMC4079202; doi:10.1093/gbe/evu095)

(A) *T2R* tree

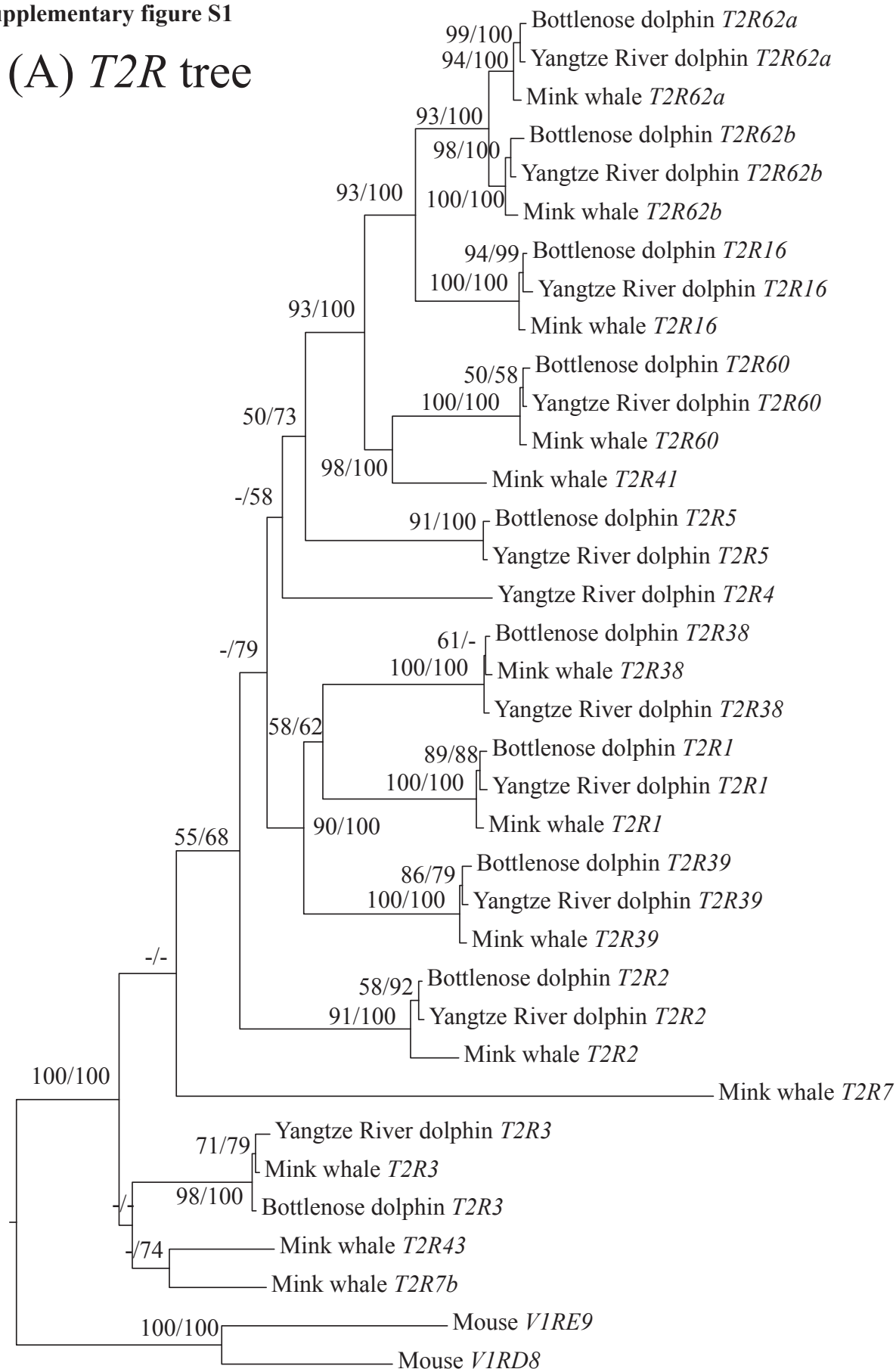

(B) *Pkd2l1* tree

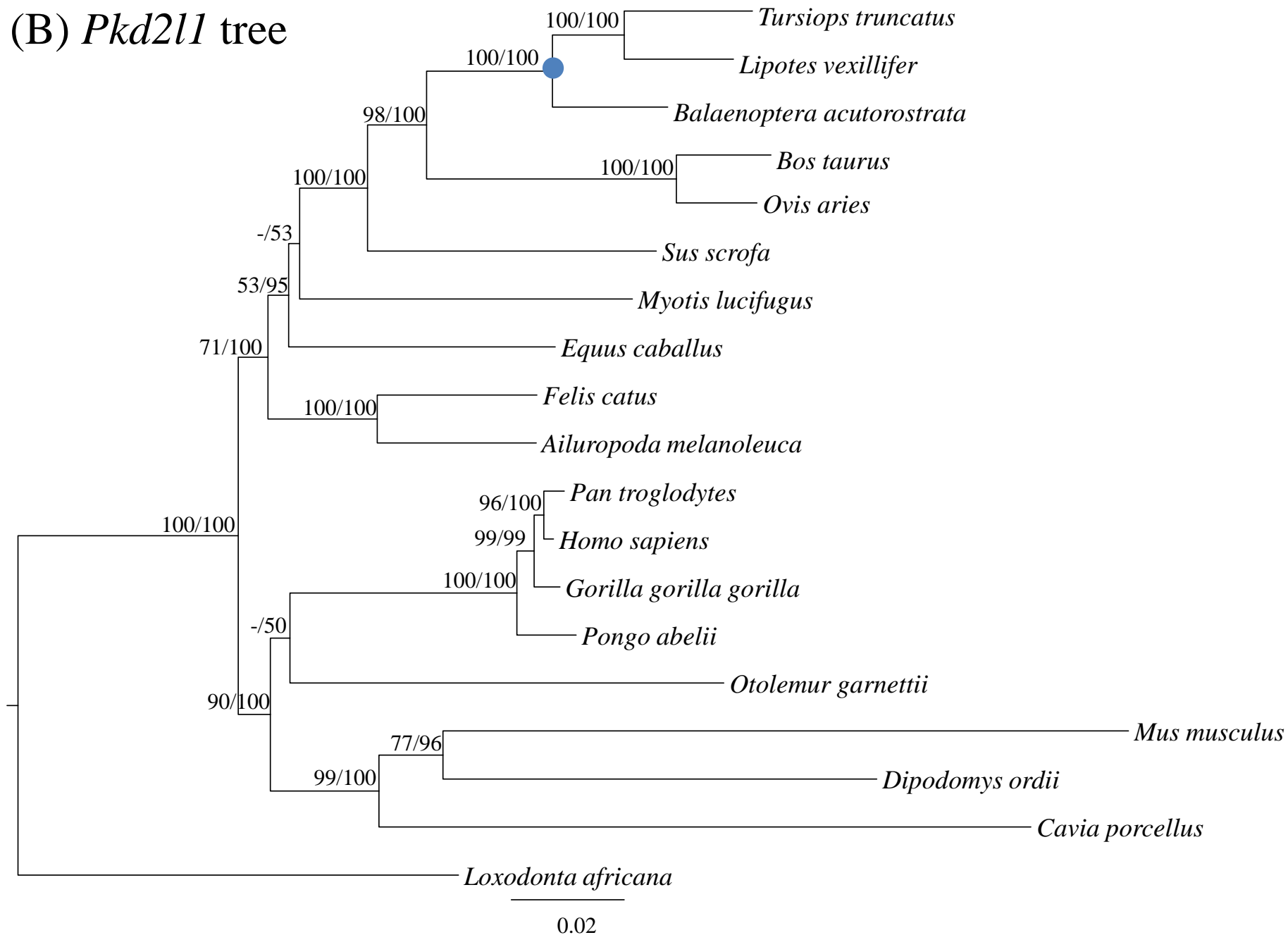

(C) *Trpm5* tree

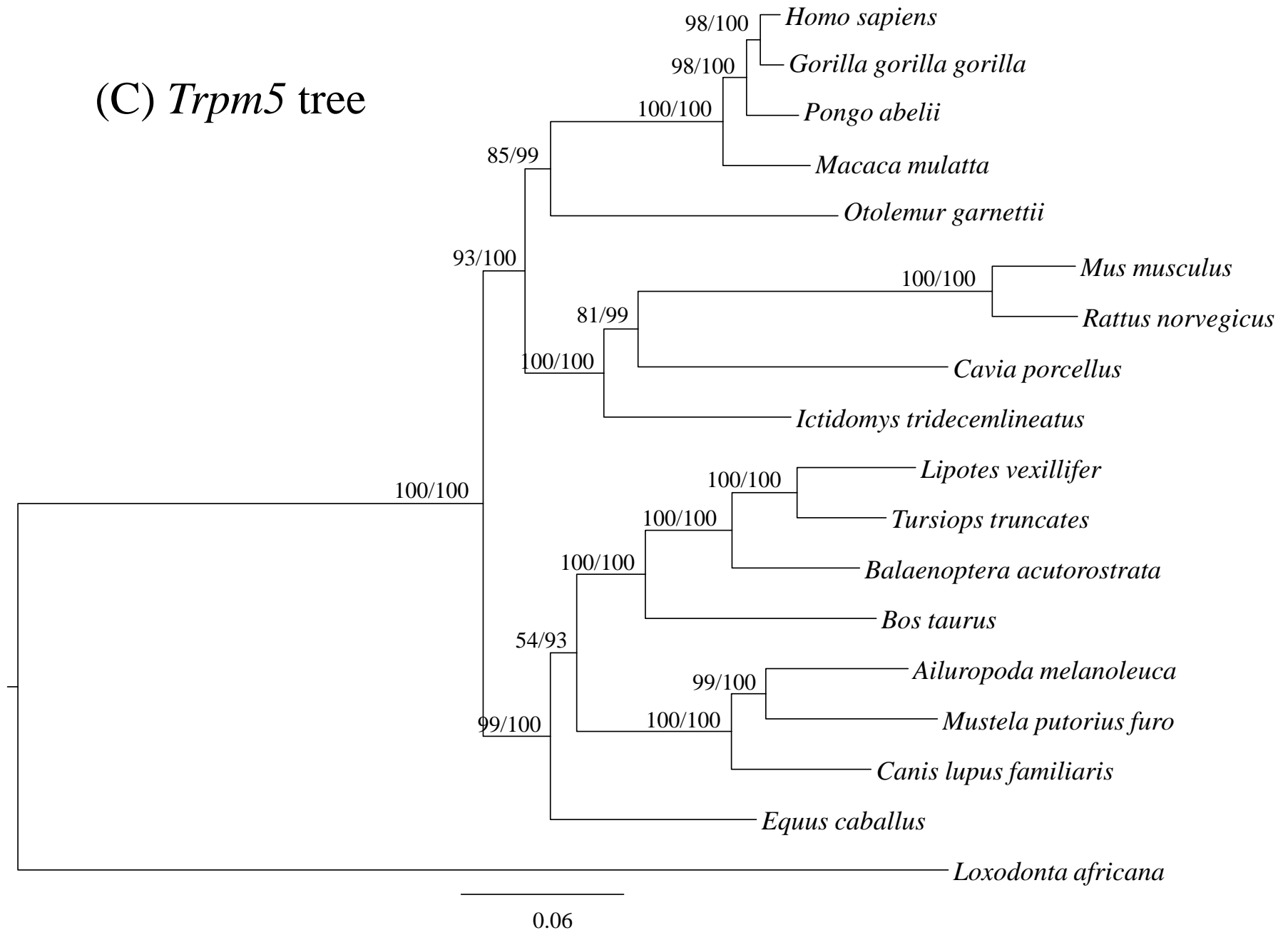

(D) *Plcb2* tree

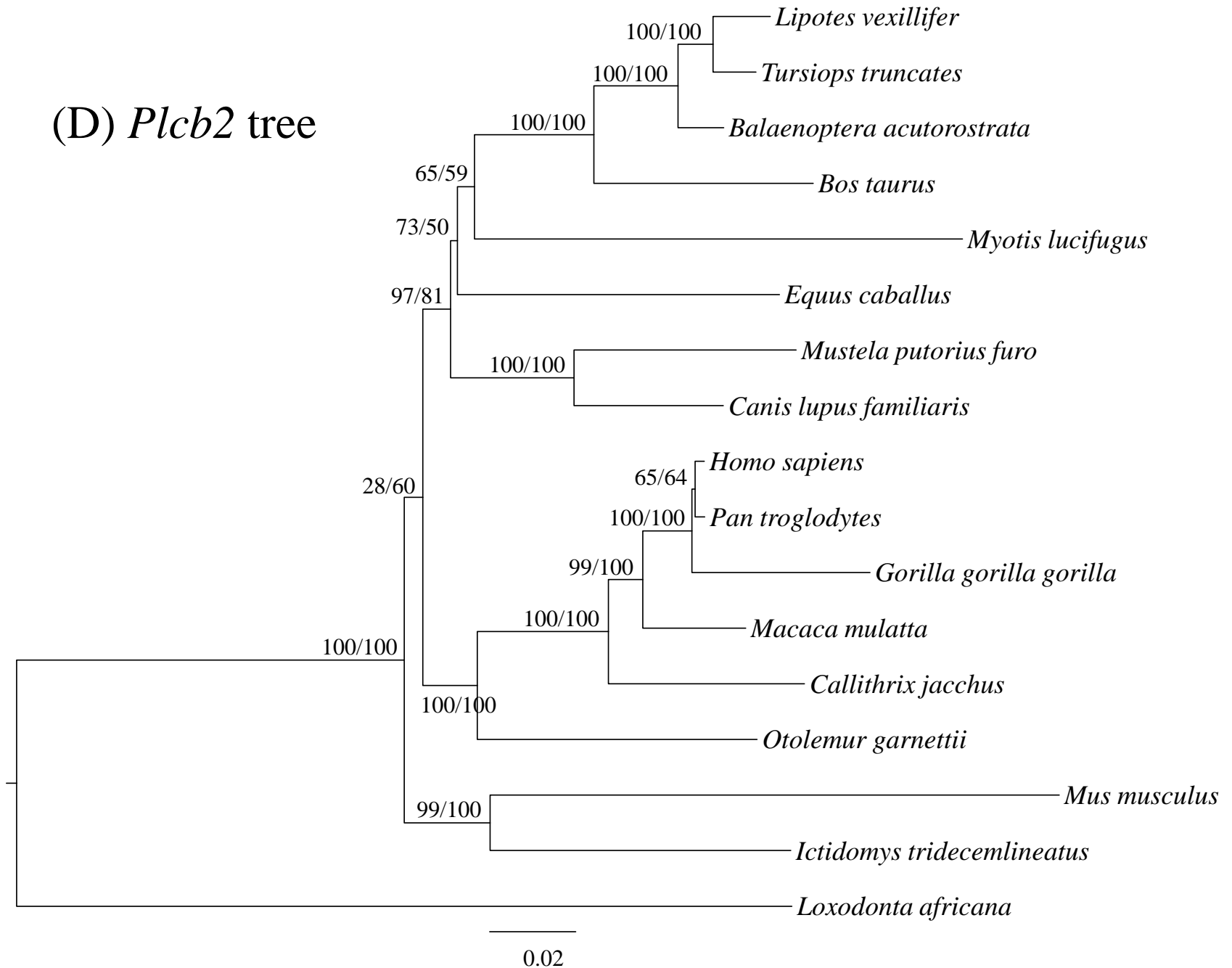

Supplement: Supplementary Data [file supp_evu095_suppl_data.zip › fig._S1_submitted.pdf]
